# Whole Genome Insights into Genetic Diversity, Introgression, and Adaptation of Hunan Cattle

**DOI:** 10.3390/ani15091287

**Published:** 2025-04-30

**Authors:** Yushan Li, Jianbo Li, Hongfeng Duan, Ao Sun, Renke Hu, Shuai Gao, Baizhong Zhang, Bizhi Huang, Chuzhao Lei, Kangle Yi

**Affiliations:** 1 Hunan Institute of Animal and Veterinary Science, Changsha 410130, China; alysali_51@163.com (Y.L.); ljbljb12@163.com (J.L.); dhfybf2004@163.com (H.D.); kkkkanty@163.com (A.S.); hurenke321@hunaas.cn (R.H.); 13975894274@163.com (S.G.); zhangbz6181@163.com (B.Z.); 2 Key Laboratory of Animal Genetics, Breeding and Reproduction of Shaanxi Province, College of Animal Science and Technology, Northwest A&F University, Yangling 712100, China; 3 Yunan Academy of Grassland and Animal Science, Kunming 650212, China; hbz@ynbp.cn

**Keywords:** adaptation, introgression, whole-genome sequencing, Hunan cattle, parental lineage, ROH classification

## Abstract

Hunan Province, located in Central-South China, has a hot and humid climate, which has shaped the unique characteristics of its cattle. In this study, we analyzed the genomic diversity of 110 indigenous Hunan cattle using whole-genome sequencing and found that they have a mixed ancestry of indicine and taurine. By grouping the cattle based on their collection regions (western, central, southeastern, and southern Hunan), we used an unsupervised three-component Gaussian model to classify the runs of homozygosity (ROH) and calculated the genomic inbreeding coefficient based on runs of homozygosity (FROH) to assess inbreeding levels. The results showed that western Hunan cattle had the highest level of hybridization, while southern Hunan cattle had the lowest. Through selective sweep analysis, we identified candidate genes and pathways related to environmental adaptation and homeostasis. Notably, the *SLC5A2* gene showed strong selection signals across all four regions and exhibited a distinct haplotype compared to other referenced cattle breeds. Additionally, we detected introgression from wild species into Hunan domestic cattle and analyzed their Y-chromosome haplotypes.

## Simple Summary

This study focused on the population structure and genetic characteristics of Hunan cattle using whole genomes of 110 individuals from four regions in Hunan. The results indicated that Hunan cattle have a mixed ancestry of taurine and indicine cattle, with high genetic diversity. Several candidate genes under selection associated with desirable traits were identified. Additionally, the study clarified the introgression of wild cattle into domestic Hunan cattle and highlights functionally relevant introgressed regions. These findings suggested that Hunan cattle possess unique genetic characteristics, such as disease resistance, hot environmental adaptability, and high-quality meat; made a substantial contribution to the development of reasonable breeding strategies and resource conservation efforts of Hunan indigenous cattle; and laid a foundation for ensuring their continued contribution to the economy and cultural heritage of the region.

## 1. Introduction

The genus *Bos* includes *Bos javanicus*, *Bos gaurus*, *Bos frontalis*, *Bos sauveli*, *Bos grunniens*, and *Bos taurus*. The domestic cattle (*Bos taurus*) gave rise to two distinct but cross-fertile cattle subspecies, taurine cattle (*B. t. taurus*) and indicine cattle (*B. t. indicus*). Domestic cattle in China mainly consist of Bos taurus taurus and Bos taurus indicus, which diverged around 200,000–300,000 years ago [1]. Archeological evidence suggests that the taurus was domesticated about 10,000 years ago and was introduced to China primarily through the Xinjiang region of northern China or the Eurasian steppe. The indicus were domesticated in the Indus Valley about 8500 years ago. Archeological and genetic studies suggest that, with human migration, the Chinese indicine may have descended from the Indian indicine cattle through migration in the southeastern coastal region. Furthermore, some archeological evidence also shows that other Asian bovine species have introgressions into Chinese cattle, especially southern Chinese indicine cattle [2]. This introgression has had a significant impact on their adaptation to the environment [3].

Hunan, located in the central-southern part of China, has a hot and humid climate, where the primary cattle breed is indicine. The local cattle are highly adaptable to these unique environmental conditions, demonstrating resilience to rough feed and strong disease resistance. Many studies have shown their unique characteristics. For instance, cattle from the Loudi area in central Hunan have been shown to have developed stronger renal functions to adapt to the humid and hot climate [4]. Cattle sourced from the Xiangxi Cattle Engineering Technology Center in western Hunan possess superior traits, such as disease resistance, environmental adaptability, and high-quality meat [5]. The Chaling cattle in southeastern Hunan are characterized by their ability to adapt to hot environments and their good meat quality [6]. Meanwhile, cattle in southern Hunan have been proven to have a pure indicine lineage, along with strong heat resistance and immune response capabilities. Furthermore, the cattle in this region also presented a distinct introgression from banteng and gaur, which contributed to the rapid adaptation to the local environment [7].

Having gone through both natural and artificial selection, a notable phenomenon is that cattle from different regions of Hunan exhibit diverse traits. In this study, we carried out comprehensive whole-genome sequencing on 110 cattle from four regions in Hunan. Among them, 93 cattle were sourced from other studies. Since previous research indicated that cattle from southeastern and western Hunan had a distinct mixed lineage, we added 17 new samples collected from these two areas to further verify this finding. We aimed to explore the unique genomic characteristics and phylogeographic patterns of the diversity of Hunan cattle by using the largest Hunan cattle genome dataset available to date. By exploring the population structure, genomic variation, selective sweeps, and introgression from other bovine species, we identified the unique adaptive features of indigenous Hunan cattle. Additionally, we also investigated the paternal lineage of Hunan cattle by comparing them with the reference genome assembly (ARS-UCD 1.2).

## 2. Materials and Methods

### 2.1. Sample Collection and Sequencing

There were 110 cattle collected from southeastern Hunan (*n* = 20), central Hunan (n = 36), western Hunan (*n* = 34) and southern Huan (*n* = 20) in the Hunan province, China. Among the 110 cattle, 17 samples were new in this study. We collected their ear tissue samples and use a standard phenol/chloroform-based protocol to extract the genomic DNA. We used Illumina NovaSeq 6000 at Novogene Bioinformatics Institute, Beijing, China, to sequence data with paired-end libraries and had an average read length of 150 bp [8]. Other Hunan cattle were collected from different projects (Table S1). To more clearly show the ancestral components of these Hunan cattle, we chose 135 samples from other studies in different and testified groups. They were Chinese indicine (*n* = 33), Indian indicine (*n* = 26), East Asian taurine (*n* = 22), Eurasian taurine (*n* = 25), and European taurine (*n* = 29) (Table S1). Finally, a total of 245 samples were used in this study.

### 2.2. Sequence Processing and SNP Calling

The first step was qualifying the raw data following the 1000 Bull Genomes Project Run 8 guideline (http://www.1000bullgenomes.com/ (accessed on 11 November 2023)) to obtain the standard-compliant data for analyzing. ARS-UCD1.2 was used as the reference genome to qualify the raw reads data by trimmomatic [9], the clean reads were aligned by BWA-MEM (v0.7.13-r1126) [10] with default parameters, and potential duplicated reads were filtered using “MarkDuplicates” in the Picard tools (http://broadinstitute.github.io/picard (accessed on 3 February 2024)). Then, we used the modules of “HaplotypeCaller”, “GenotypeGVCFs”, and “SelectVariants” in Genome analysis toolkit 3.8 (GATK) [11] to call the SNP. The “VariantFiltration” module with hard filtering parameters “QD < 2.0, FS > 60.0, MQ < 40.0, MQRankSum < −12.5, ReadPosRankSum < −8.0, and SOR > 3.0” was used to filter the raw SNPs and the mean sequencing depth of variants (all individuals) “<1/3× and >3×”. The SNPs in different regions were functionally annotated by ANNOVAR [12] with the annotation file (GCF_002263795.1_ARS-UCD1.2_genomic.gff) of the B. taurus reference genome.

### 2.3. Population Genomic Analysis

Plink 1.9 [13] was used to transfer VCF to plink format and calculate the matrix of pairwise genetic distances. The neighbor-joining (NJ) tree was constructed with the non- linkage disequilibrium (LD) sites extract by Plink, aligned using MEGA v10.0 [14], and finally visualized with iTOL (https://itol.embl.de/ (accessed on 10 April 2024)). In addition, the SNPs in high levels of pairwise linkage disequilibrium (LD) with the parameter (–indep-pairwise 50 5 0.2) were pruned for principal component analysis (PCA) and ADMIXTURE analysis. The smartPCA program of ELGENSOFT v5.0 package [15] was used to carry out the result of PCA. Population structure analysis was performed by ADMIXTURE v1.3 [16] with the kinship (K) parameter set from 2 to 8 for each possible group number.

### 2.4. Detection of Genetic Diversity

Including the four regions of Hunan cattle and the reference samples, there were nine groups of cattle. The smartpca program with EIGENSOFT v.4.2 [15] was used to estimate the *F*_ST_ between the 9 groups. The nucleotide diversity of each group was investigated by VCFtools [17], keeping a window size of 50 kb and a step size of 20 kb. PopLDdecay [18] was used to calculate the physical distance between SNPs with default parameters.

### 2.5. Extract, Auto-Classify and Analyze ROH Region

Beagle [19] was used to phase the VCF file with the parameter “gprobs = true niterations = 10 nthreads = 48”. The SNPs with a dominance ratio (DR) > 0.9 were extracted by Python script. Plink was used to extract ROH regions with the parameters “--homozyg-gap 1000; --homozyg-kb 100; --homozyg-snp 200; --homozyg-window-het 1; --homozyg-window-snp 100; --homozyg-window-threshold 0.05”. Mclust v.3 package (https://mclust-org.github.io/mclust/ (accessed on 6 August 2024)) in R was used for unsupervised three-component Gaussian fitting of the ROH length distribution to determine the specific boundaries of three types of ROH in the 9 groups cattle in this study. The genomic inbreeding coefficient based on runs of homozygosity (FROH) is defined as the proportion of the genome covered by runs of homozygosity (ROH) relative to the total length of the autosomal genome. It is calculated using the following formula: FROH = L(ROH)/L(Autosomes) where L(ROH) represents the total length of all ROH detected in a sample and L(Autosomes) denotes the total length of the autosomal genome covered by the analyzed SNPs [20].

### 2.6. Selective Sweep Identification

Both the nucleotide diversity (θπ) and integrated haplotype score (*iHS*) were applied to detect the positive signatures in Hunan cattle. θπ is a measure of genetic variation within a population, which reflects the average number of nucleotide differences per site between any two randomly chosen DNA sequences. It is often used to identify regions with reduced diversity that may indicate selective sweeps, and we set a 50 kb sliding window. Candidate regions were selected by calculating a cutoff value based on the inverse survival function of a normal distribution, whose values are in the top 1%. On the other hand, the integrated haplotype score (*iHS*) detects signals of recent positive selection by comparing the decay of extended haplotype homozygosity (EHH) around derived and ancestral alleles. The proportion of SNPs with |*iHS*| ≥ 2 was calculated by Selscan v2.0 [21] with nonoverlapping 50 kb windows and 20 kb steps. The top 5% regions were considered as candidate regions. The overlapped genes on these regions detected in these two methods were considered as candidate genes.

To better understand the function of these candidate genes, we applied KOBAS 3.0 (http://bioinfo.org/kobas/ (accessed on 3 October 2024)) and selected the p-adjusted < 0.05 pathways in Kyoto Encyclopedia of Genes and Genomes (KEGG) and Gene Ontology (GO). We even calculated the *F*_ST_, Tajima’s *D*, and nucleotide diversity information of candidate genes by using VCFtools [17].

### 2.7. Introgression Analysis

RFMix v2.02 [22] was used to identify introgressed regions of banteng and gaur in Hunan cattle with *D* statistics [23]. To avoid the bias, pure indicine cattle, taurine cattle, banteng, gaur, and other wild cattle species (Table S9) were served as the reference group with a |Z score| > 3 according to the *D* statistics. Furthermore, we calculated the incomplete lineage sorting (ILS) [24]. Here, r is the recombination rate per generation per base pair (bp) in indicine cattle, m is the length of the introgressed tract, and t represents the length of other wild species (bangteng and gaur) since divergence [2]. The expected length of a shared ancestral sequence is L = 1/(r × t) = 206.52 bp. The probability of a length of at least m is 1-GammaCDF (m, shape = 2, r = 1/L), in which GammaCDF is the gamma distribution function [3]. We applied the probability of ILS < 0.05 to filter the results of RFMix, confirming the introgression ratio of four regions in Hunan. Then, we used U20 _Indian indicine, Hunan cattle, Banteng and Gaur_ (1%, 20%, 100%) to identify highly frequent introgressed sites with 50 kb windows and 20 kb steps [25]. The execution pattern was as follows: banteng or gaur had a particular allele at a frequency of 100%, while the frequency was less than 1% in indicine cattle but greater than 20% in Hunan cattle. We detected the introgressed segments from banteng and gaur to Hunan cattle, respectively, and overlapped these introgressed segments to find the overlapped genes. The results of the U20 statistics were used to determine the functions and complex pathways by using a python script. The likelihood tree visualized by FigTree v1.4.4 (http://tree.bio.ed.ac.uk/software/figtree/ (accessed on 25 July 2023)) with the haplotype map showed the regions of differentiation, and it was used to confirm the results and make the analysis more rigorous [26,27].

### 2.8. Paternal Analysis

We select the X-degenerate region containing single-copy genes within the male-specific region of the Btau_5.0.1 Y-chromosome reference sequence (GCF_000003205.7), involving 44 Hunan cattle and 10 reference individuals (Table S10). After removing heterozygous sites and sites with missing genotypes in 10%, the remaining SNPs were extracted for analysis. Haplogroup trees were constructed from FASTA-formatted sequences using maximum likelihood (ML) methods.

## 3. Results

### 3.1. Detecting and Classifying the Single Nucleotide Polymorphism (SNP)

**Figure 1:**
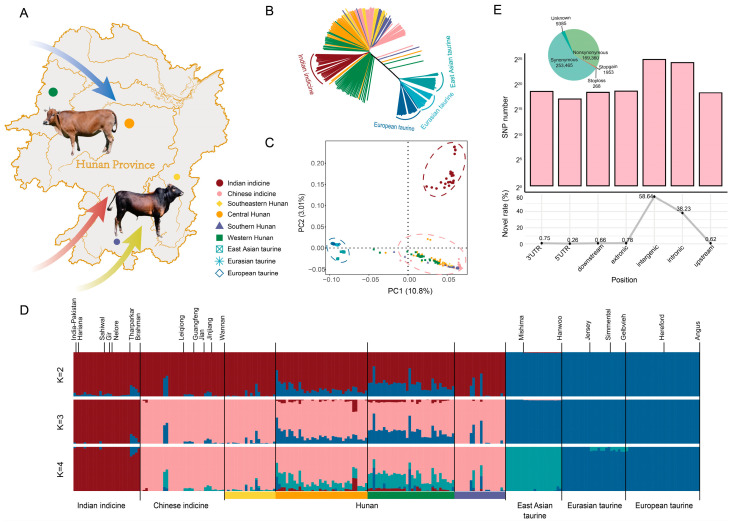
Population genetic analysis of Hunan cattle in comparison to several possible ancestral breeds. (**A**) Sample collection map of Hunan cattle in this study. Size of the circle represents the number of samples. The blue, red, and yellow arrows represent the migration routes of taurine cattle, indicine cattle, and other wild species in China, respectively. The indicated directions are approximate [3,8,28]. (**B**) The neighbor-joining phylogenetic tree of the 245 domesticated cattle. The colors reflecting different groups of sampling are the same in the PCA. (**C**) Principal component analysis (PCA) showing PC1 against PC2. (**D**) Model-based clustering of cattle breeds using ADMIXTURE (K = 2, 3, 4). Each vertical bar representing an individual is colored by different ancestral breeds and labeled with the breed name. (**E**) Functional classification of the detected SNPs.

The nine groups, which composed of 245 cattle, were selected for genome re-sequencing analysis (Table S1), representing Indian indicine, Chinese indicine, East Asian taurine, Eurasian taurine, European taurine [1], and Hunan. These were collected by four local populations in Hunan province (Figure 1A). A total of 110 cattle in Hunan province served as the target group in this study, with an average sequence coverage of ~11.28X and a mapping rate of 99.73%, using ANNOVAR [12] to annotate SNPs: 58.64% were located in intergenic regions and 38.23% in intronic regions whilst the following were located in the upstream and downstream regions (1.28%) and UTR regions (1.01%). Only 0.78% of SNPs are located in the exon regions and they divide into five types with 253,465 synonymous and 169,360 nonsynonymous (Figure 1E) (Table S2).

Principal component analysis (PCA) showed three cluster of 245 sampling resequencing data, representing taurine, Indian indicine, and a big cluster of Hunan cattle and Chinese indicine (Figure 1C). The PC1 explained 10.8% variation and departed *B. taurus* from *B. indicus*. The second PC (PC2) explained 3.01% of the total variation with geographically separated Chinese indicine and Indian indicine. Hunan cattle as the target group were classified into Chinese indicine. To further explore the genetic relationships among the target group and other several possible ancestral breeds, we used a 245 × 3,422,976 counts matrix to construct the phylogenetic tree using the neighbor-joining way (Figure 1B) and analysis by ADMIXTURE. The cattle breeds separate into *Bos taurus* and *Bos indicus* ancestry (K = 2), where the Chinese indicine and Indian indicine depart (K = 3), and when K = 4 with the least CV error Hunan cattle exhibit a mixture of up to four ancestral lineages. At the same time, the East Asian taurine separated from other taurine. From the structure result, the cattle of four regions all had a mixed lineage of taurine and indicine, and the southeastern and the southern Hunan cattle had a purer lineage of Chinese indicine compared with other two regions (Figure 1D).

### 3.2. Detecting, Classifying and Genomic Variation Analysis of the SNPs

**Figure 2:**
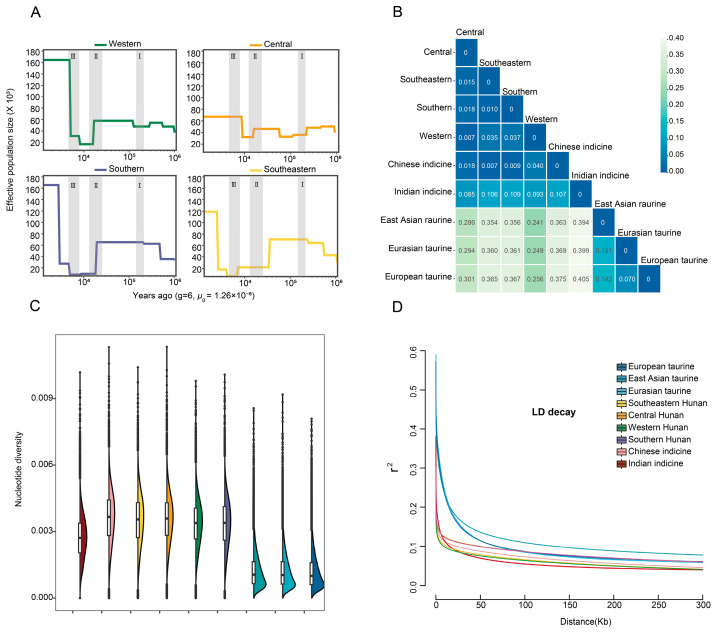
Population genetic analysis of Hunan cattle in comparison to several possible ancestral breeds. (**A**) Demographic reconstruction of effective population size (Ne) in four Hunan groups. From right to left are the second glacial period (I), the last glacial maximum (II), and the Early Holocene Optimum (III). (**B**) Mean *F*_ST_ values between pairwise groups. (**C**) Nucleotide diversity of different groups. The colors reflecting different groups of sampling are same as the LD plot. (**D**) Genome-wide average LD decay is estimated from each group.

Past demographic reconstruction using SMC++ [29] indicated the changes in effective population size (*Ne*) in four Hunan regions. Western and southern Hunan have a similar effective population size pattern, which was relatively stable in the Second Pleistocene glacial period, gradually declined in the last glacial medium, and increased in the Early Holocene optimum. Central Hunan only had a gradual decline in the last glacial medium while southeastern Hunan had a decline in the Early Holocene optimum (Figure 2A) [1,8]. Genetic distance measurements among these group were assessed using an *F*_ST_ matrix, revealing variability ranging from 0.007 to 0.405. There is a close distance within Hunan cattle, whilst Hunan cattle show subtle differences with Chinese indicine and more differences with Indian indicine, and show notably higher genetic differentiation from *Bos taurus* (Figure 2B). The nucleotide diversity analysis of these cattle breeds revealed that *Bos indicus* has a higher average nucleotide diversity, especially in Chinese indicine (3.61 × 10^−3^) and Hunan cattle, respectively, in the southern Hunan (3.36 × 10^−3^), the western Hunan (3.36 × 10^−3^), the southeastern Hunan (3.50 × 10^−3^) and the central Hunan (3.56 × 10^−3^), whilst Indian indicine (2.71 × 10^−3^) are lower than them but are higher than taurine. Among all taurine, European cattle is the lowest one (1.19 × 10^−3^), while Eurasian taurine (1.22 × 10^−3^) and East Asian taurine (1.23 × 10^−3^) are higher (Figure 2C). The LD plot showed *Bos indicus* has a faster decay compared with Bos taurus when the physical distance of SNP is less than 10 KB. The samples in central and western regions have a faster decay than the purer samples in the southern and southeastern regions of Hunan (Figure 2D).

### 3.3. Run of Homozygosity (ROH) Classification, Calculation, and Inbreeding

**Figure 3:**
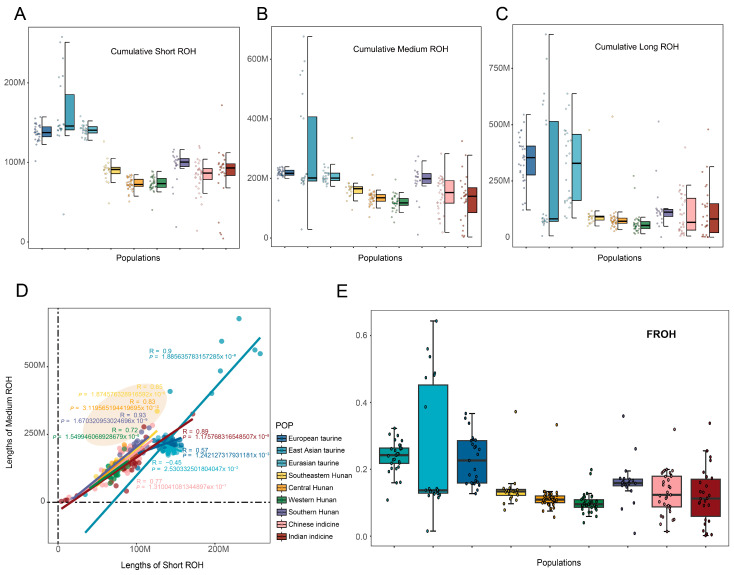
The analysis of ROH length and correlation. (**A**–**C**) The cumulative length of short, medium, and long ROH based on the module of the unsupervised three-component Gaussian. (**D**) Correlation between the cumulative lengths of short ROH and medium ROH. The orange region is Hunan cattle. (**E**) FROH in different groups. The color represents different groups.

The analysis of run of homozygosity (ROH) can reveal the different inbreeding coefficients of different groups. Unsupervised three-component Gaussian fitting can auto sort the length of ROH into short, medium and long level, and different groups had their unique threshold value (Tables S3 and S4). We calculated the total length of nine groups at each level and counted the number of ROHs on three levels. Although the number of ROH in different groups may be affected by the amount of samples, the length of three levels is proportional to the number (Figure S1). The *Bos taurus* both have higher cumulative length in three levels, especially compared with the European taurine. Among the four regions in Hunan, the southern Hunan has the highest cumulative length, followed by the southeastern Hunan, the central Hunan, and the western Hunan which is the least (Figure 3A–C). To explore the correlation between short ROHs and medium ROHs, as well as short ROHs and long ROHs across all groups, the results showed that short ROHs were more strongly correlated with medium ROHs but had weaker correlations with long ROHs (Figure 3D and Figure S2). Finally, we used the length of ROH segments to calculate inbreeding coefficients of each group. Among the four Hunan groups, the southern Hunan had the highest inbreeding coefficient while the western Hunan had the lowest (Figure 3E).

### 3.4. Selection Signatures in Hunan Cattle

**Figure 4:**
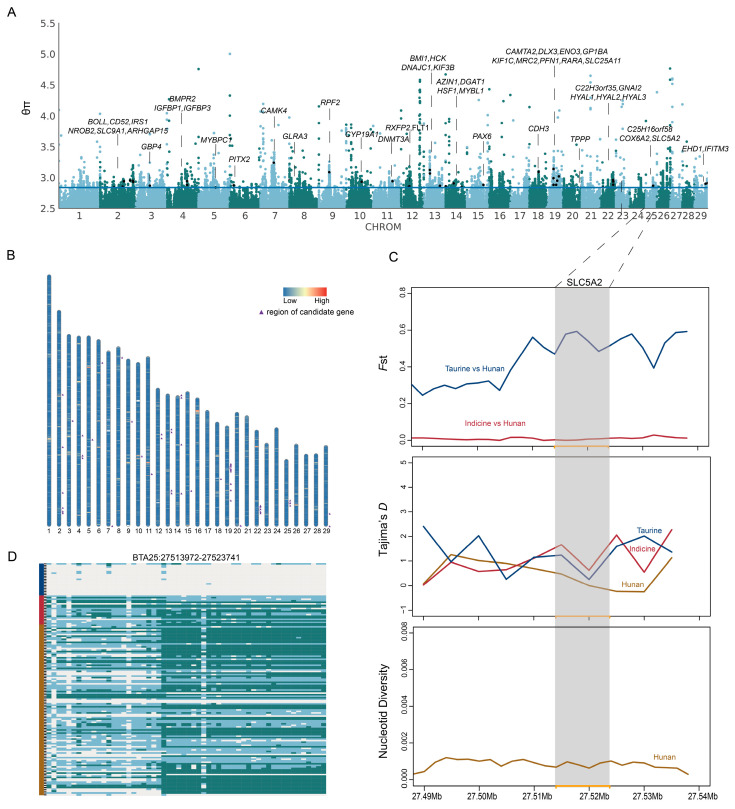
Positive signatures in Hunan cattle. (**A**) Manhattan plot of selective sweeps by θπ. The marked genes were candidate genes. (**B**) The mixture of the regions with |*iHS*| > 2. Low intensity is represented in blue and high intensity in red. The purple markers indicate the locations of the candidate genes, which were the same as the θπ method. (**C**) *F*_ST_, Tajima’s *D*, and nucleotide diversity plot of the *SLC5A2* gene. The gray-shaded boxes represent the region of *SLC5A2*. (**D**) Haplotype patterns were plotted by heatmap. The dark blue represents taurine, dark red represents indicine, and brown represents Hunan cattle.

The four regions in Hunan consist of a characteristic group. To find out the special functional gene in this group, we applied the integrated haplotypes score (*iHS*) and nucleotide diversity analysis (θπ) methods. A total of 1669 (*iHS*) and 2044 (θπ) genes with selection signatures in Hunan cattle were found, 243 genes were overlapped in these two methods (Tables S5–S7), and 49 of these were identified as candidate genes (Figure 4A,B and Figure S3). These candidate genes were related to reproductive function (*BOLL*, *CYP19A1*, *DLX3*, *HCK*, *MYBL1*, *FLT1*), immune response (*CD52*, *ENO3*, *GNAI2*, *HYAL2*, *IFITM3*, *IGFBP3*), meat quality (*DNMT3A*, *MYBPC1*, *NROB2*, *PAX6*, *IRS1*), hot shock (*DNAJC1*, *HSF1*), the balance of ionic concentrations (*EHD1*, *SLC25A11*, *SLC5A2*, *SLC9A1*), and others (Table S7). The enrichment results also showed that the genes were enriched in immune responses (GO:0046677), UV-B response (GO:0071493, GO:0030214), and cartilage development (GO:0051216) (Table S8, Figure S4). Among these candidate genes, *SLC5A2* showed a stronger signature in Hunan cattle, which was proved in Tajima’s *D*. Although there was a lower *F*_ST_ value between indicine and Hunan cattle, the haplotype patterns were different in these two groups (Figure 4C,D).

### 3.5. The Introgressed Events Form Other Wild Species

**Figure 5:**
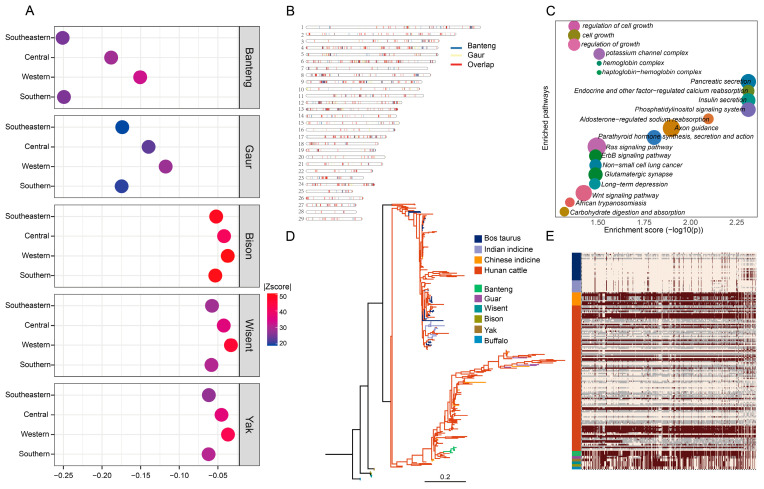
Genome-wide introgression from wild Bos species into Hunan domestic cattle. (**A**) Alle sharing of f4 pop (Indian indicine, Hunan cattle, wild, buffalo) between Hunan cattle and wild species. The circle represents |Z-score|. (**B**) Map of introgressive segments from banteng and gaur in Hunan cattle, the blue lines represent the segments from banteng, yellow lines represent gaur, and red lines represent the overlap segments from these two species. (**C**) The KEGG and GO pathways from the genes on the overlap segments from banteng and gaur into Hunan cattle. (**D**) Phylogenetic trees were constructed using the haplotype sequences of BTA6: 70.14 Mb–70.22 Mb region. (**E**) Haplotype patterns were built by SNPs in the region of BTA6: 70.14 Mb–70.22 Mb region. Color of the blank represents different populations.

We calculate the introgression from five wild species (banteng, gaur, yak, wisent, bison) into Hunan cattle, using *D*-statistics to confirm it. Compared with the Indian indicine, the wild species had more shared segments with Hunan cattle, with a |*Z*-score| > 3, and banteng and gaur had the most shared segments with Hunan cattle (Figure 5A) (Table S11). The U20 shared segments from banteng and gaur were in different chromosome regions, and a lot of these segments are overlap introgressive segments (Figure 5B). Using a *p* < 0.05 cutoff for the frequencies of introgression alleles in the Hunan cattle genomes (U20 _Indian indicine, Hunan cattle, Banteng and Gaur_ (1%, 20%, 100%)), 1060 candidate genes were shortlisted and enriched in KEGG pathways and GO terms, which were associated with immunity, cell growth, hydro-salinity balance, and adaptability to the environment (Figure 5C) (Table S12). We found a region on the Bos taurus autosome (BTA) 6 (70.14 Mb–70.22 Mb) which represents obvious introgressions from the wild species into Hunan cattle, which contained a part of the *KIT* gene (BTA6:70166692-70254049). To further understand if this region is from introgression with wild species or not, we constructed a likelihood tree and haplotype pattern heatmap (Figure 5D,E).

### 3.6. Parental Analysis

**Figure 6:**
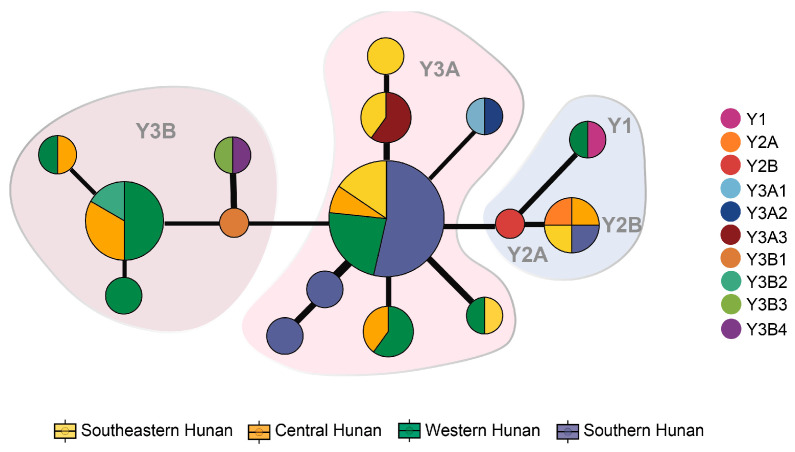
Y-chromosome phylogenies. The circular area is proportional to the sample size. MJ network of Y-chromosome haplotypes in four regions of Hunan male cattle. The blue shadow covers the haplotype in taurine cattle, while the pink shadow represents indicine haplotype. Different levels of pink were used to differentiate Y3A and Y3B.

The results of Y-chromosome haplotypes of the 44 male cattle revealed the division of Hunan domestic cattle into two major parental lineages, Y3A3 and Y3B2, which are two indicine cattle sub-haplotypes. Moreover, the central Hunan group has one individual related to the taurine cattle haplotype (Y1 haplotype) while the other three groups have one individual related to another taurine cattle haplotype, respectively (Y2A sub-haplotype) (Figure 6). The specific distributions were as follows: western Hunan region (*n* = 15), one bull with Y1 haplotype, seven bulls with the Y3B2 sub-haplotype, and seven bulls with the Y3A3 sub-haplotype; central Hunan region (*n* = 7), one bull with the Y2A sub-haplotype, two bulls with the Y3A3 sub-haplotype, and four bulls with the Y3B2 sub-haplotype; southeastern Hunan region (*n* = 9), one bull with the Y2A sub-haplotype, and eight bulls with the Y3A3 sub-haplotype; southern region (*n* = 13), one bull with the Y2A sub-haplotype, and twelve bulls with the Y3A3 sub-haplotype (Table S10) [3].

## 4. Discussion

In the context of global climate change and animal breeding, natural selection and human-made selection play a crucial role. As the climate changes, specific environmental pressures emerge. Under these circumstances, within a population of animals certain genetic variations that are beneficial for survival and reproduction in the new environmental conditions start to come into play. These adaptive traits then confer a selective advantage to the individuals possessing them, allowing them to better thrive in their respective environments. As a southern province in China, previous studies reveal an interesting phenomenon that different regions of Hunan exhibit various traits, indicating that they may be located in a hybrid area of Chinese indicine cattle and taurine cattle. However, these scattered studies cannot reflect the shared selective characteristics of Hunan cattle. Different from previous studies, we collected the largest genomic dataset available at present to explore the unique genetic patterns of Hunan indigenous cattle. Based on autosomal genome-wide analyses, we first highlighted the taurine × indicine admixture characteristics of Hunan cattle, and that Chinese indicine is the dominant ancestry of Hunan cattle. The genetic distribution patterns among 110 local cattle in four regions of Hunan showed a gradual decrease in indicine ancestry from south to north and a distinct decrease in taurine ancestry from north to south. In terms of parental lineage, the cattle of four regions all have a purer indicine origin, with Y3 haplotype. However, there are some individuals contain a large proportion of components of European taurine cattle, which are the result of the blind over-introduction of local people, which will seriously lead to the loss of local cattle characteristics.

Long runs of homozygosity (ROHs) arise when identical haplotypes are inherited from each parent and thus a long tract of genotypes is homozygous. The number and length of an ROH provides insight into an individual demographic history. In the result of our study, the western Hunan cattle, which have the highest degree of hybridization, have the fewest ROHs and a higher proportion of short ROHs, while the southern Hunan cattle, with the lowest degree of hybridization, have the longest ROHs. The result of FROH showed a similar result that the western Hunan cattle have the lowest level of inbreeding, while the southern Hunan cattle have the lowest [20,29]. This congruence between the two sets of results strongly indicates that the ROH classification using the unsupervised three-component Gaussian fitting in this study tends to be a scientific method. This scientific categorization not only helps in better understanding the genetic background of these cattle but also provides valuable guidance for future livestock breeding strategies and conservation efforts in the region.

Under natural selection, certain beneficial genetic variations may gradually accumulate within a population, leading to adaptive traits that provide individuals with a selective advantage in specific environments. After selective scanning to determine whether Hunan cattle inherited adaptive advantages of ancestral populations under selection pressure, a series of candidate regions were identified in Hunan cattle, including genes related to heat tolerance, immune response, and meat quality. We finally identified the *SLC5A2* gene with a unique haplotype expression pattern in Hunan cattle, which may play a role in optimizing metabolism, maintaining kidney function, enhancing disease resistance, and adapting to the hot and humid environment by regulating glucose metabolism and ion balance [30,31]. This result can provide a reliable theoretical basis for the strong environmental adaptability of Hunan local cattle, enabling breeders to gain a deeper understanding of the genetic traits of local cattle and potentially use this knowledge in breeding programs.

Furthermore, increasing evidence suggests that gene flow occurred in the past between domestic and wild cattle in East and Southeast Asia, including banteng, gaur, yak, wisent, and bison. Migration patterns and climatic factors have played a role in the introgression of indicine cattle with wild species such as gaur and banteng, contributing to their domestication. At the same time, regional species like banteng and gaur have enriched the genetic diversity of domestic indicine cattle, improving their adaptability to different environments [3,8]. In previous studies, only the introgression tract of cattle in the southern area was calculated [7]. However, due to their purer indicine lineage, these cattle could not represent all the cattle in Hunan province. So, we used this dataset to identify the shared introgression areas in indigenous Hunan cattle. This study confirms that introgression events have significantly enhanced the genetic diversity of indigenous Hunan cattle. According to the result of *D*-statistics, we found several regions that have been introgressed from banteng and gaur into Hunan domestic cattle, which may contribute to their adaption ability to the humid area and subsequent rapid dispersal. Moreover, after analyzing by U20 statistics, the KEGG and GO enrichment results of the candidate genes shared by banteng and gaur revealed that there was distinct introgression from these two species to Hunan domestic cattle on immune system, hydro-salinity balance, and environmental adaption.

We also found several introgressed regions. One of these regions contains a *KIT* gene associated with immunity on BTA 6 (70.14 Mb–70.22 Mb), which showed a similar haplotype with the wild species and is a typical introgressed gene in cattle. Furthermore, the region on BTA 12 (73.14 Mb–73.20 Mb) contains a harbored *DNAJC3* gene relevant to heat stress, which encodes a protein that belongs to the heat-shock protein 40 (*HSP 40*) family [32]. We also found a large region on BTA 13 (51.45 Mb–53.97 Mb) which contains the *HSPA12B* gene belonging to the *HSP70* family and the *DNAJC5* gene belonging to the *HSP40* family [33]. These regions related to heat shock may prove the contribution from banteng and gaur to the adaptation ability of Hunan domestic cattle related to heat. Additionally, we found keratin associated proteins on BTA1 (5.57 Mb–5.67 Mb), which are *KRTAP13-2* and *KRTAP27-1*, and the *KRTAP* family was identified as being unique to mammals and associated with the development of hair content characteristics [34]. By analyzing these additional wild samples, we can precisely assess the contribution of other Asian bovine species to the environmental adaptation of Chinese indicine cattle. Such knowledge has practical applications in cattle breeding and conservation. And it is crucial for creating more effective strategies to protect the valuable genetic resources that are related to environmental adaptation in these cattle species.

## 5. Conclusions

In this study, we provide a theoretical basis by integrating genome analysis and archeological analysis. Results from both autosomal and sex-chromosome genome analyses indicate that the cattle in Hunan province are located in the hybrid area of Chinese indicine cattle and taurine cattle. Moreover, we identified many candidate genes with different functions through genome scanning, which enables breeders to better understand and utilize the genetic traits of indigenous Hunan cattle. Furthermore, we verified that there was distinct introgression from wild species to Hunan domestic cattle in the immune system, hydro-salinity balance, and environmental adaption. Our findings have made a substantial contribution to the development of reasonable breeding strategies and resource conservation efforts of Hunan indigenous cattle and lay a foundation of ensuring their continued contribution to the economy and cultural heritage of the region. However, the study has limitations, including the need for more comprehensive integration with multi-omics data and the lack of further validation of the candidate genes we identified.

## Data Availability

All raw sequencing data have been submitted to the NCBI SRA under the BioProject accession number PRJNA1240382.

## Supplementary Materials

The following supporting information can be downloaded at: https://www.mdpi.com/article/10.3390/ani15091287/s1.

## Abbreviations

The following abbreviations are used in this manuscript:

SNP	Single nucleotide polymorphism
WGS	Whole-genome sequencing
KEGG	Kyoto Encyclopedia of Genes and Genomes
GO	Gene Ontology
NJ	Neighbor-joining
ML	Maximum Likelihood
ROH	Runs of homozygosity
FROH	Genomic inbreeding coefficient based on Runs of Homozygosity
LD	Linkage disequilibrium
PCA	Principle component analysis
