# Supplementary material for: Whole Genome Insights into Genetic Diversity, Introgression, and Adaptation of Hunan Cattle
Source: Animals (Basel). 2025 Apr 30;15(9):1287. doi: 10.3390/ani15091287 (PMC12071150; doi:10.3390/ani15091287)
Supplement: Supplementary file 1 [file animals-15-01287-s001.zip › Supplementary Material 2.pdf]

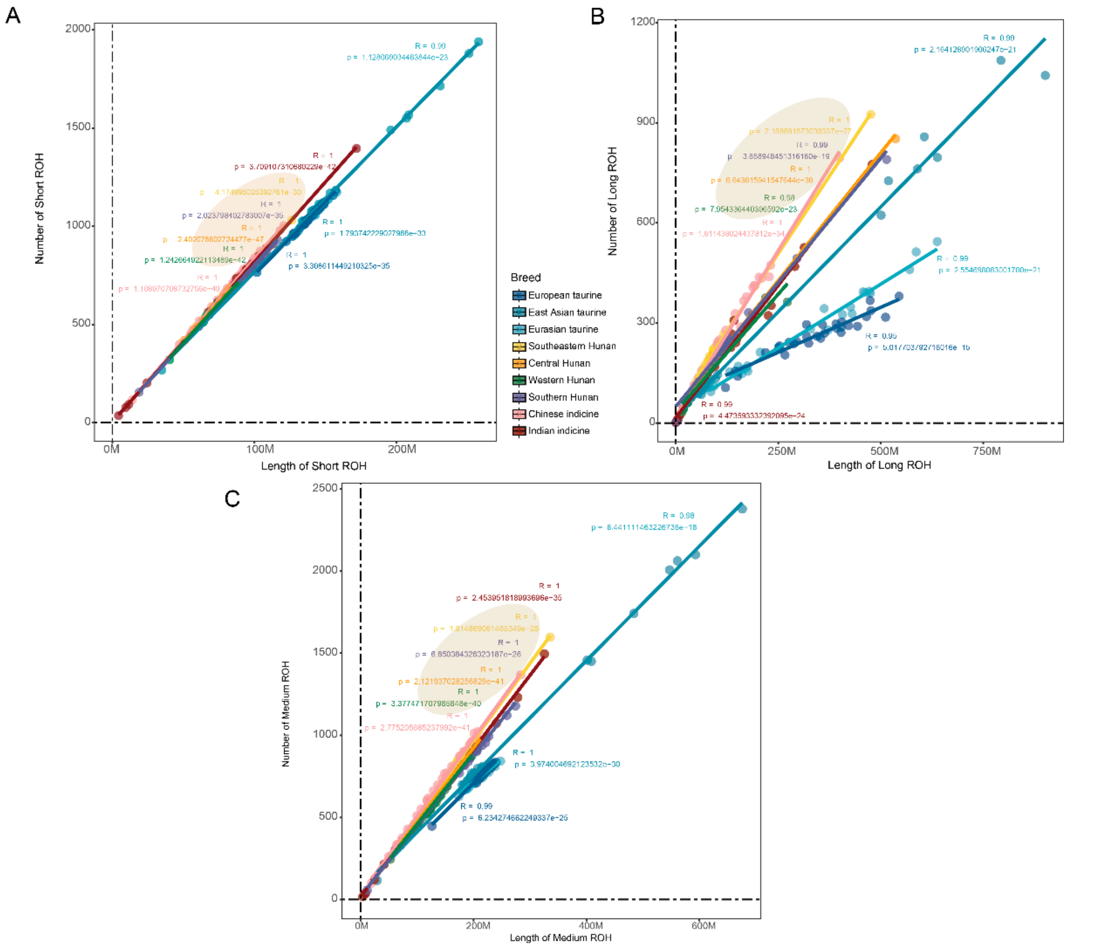

Fig. S1 The ROH levels based on unsupervised three-component Gaussian fitting. (A) The correlation of “A” level length and “A” level number. (B) The correlation of “C” level length and “C” level number. (C) The correlation of “B” level length and “B” level number.

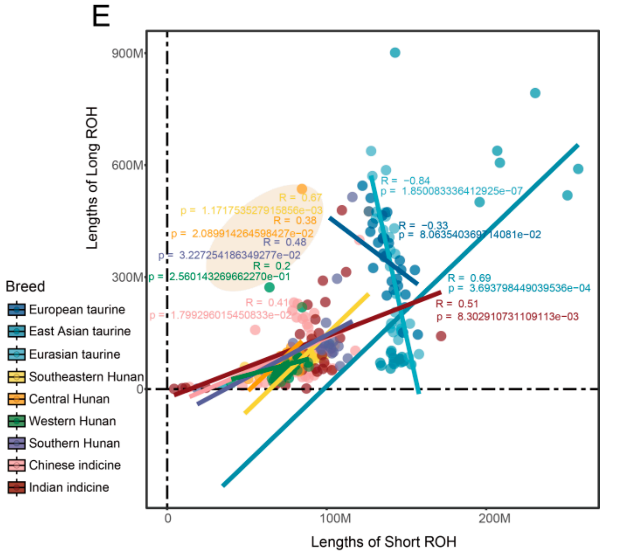

Fig. S2 Correlation between cumulative lengths of short ROH and long ROH. The orange region is Hunan cattle.

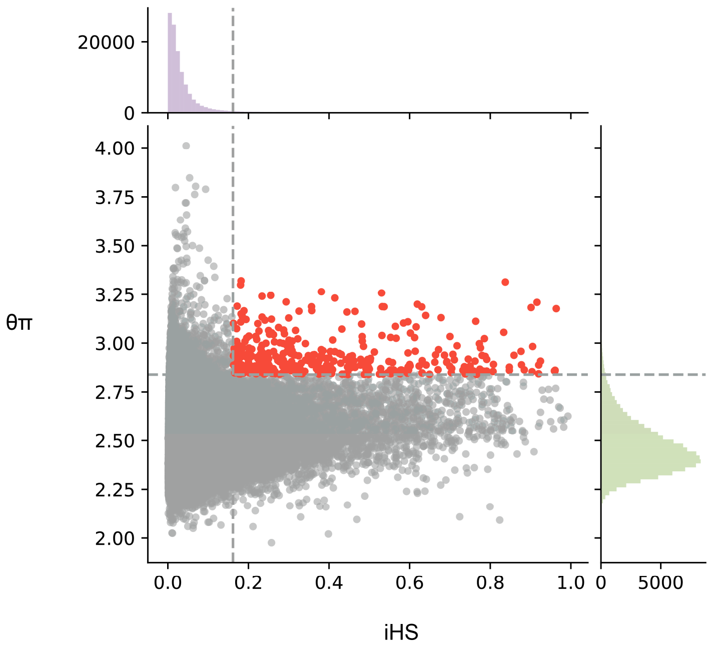

Fig. S3 Distribution of iHS and θπ. The red data points were identified as overlapping selected regions by these two methods. The gray points represented genes without overlapping in the results. The green part represented the result of θπ, while the purple represented the result of iHS.

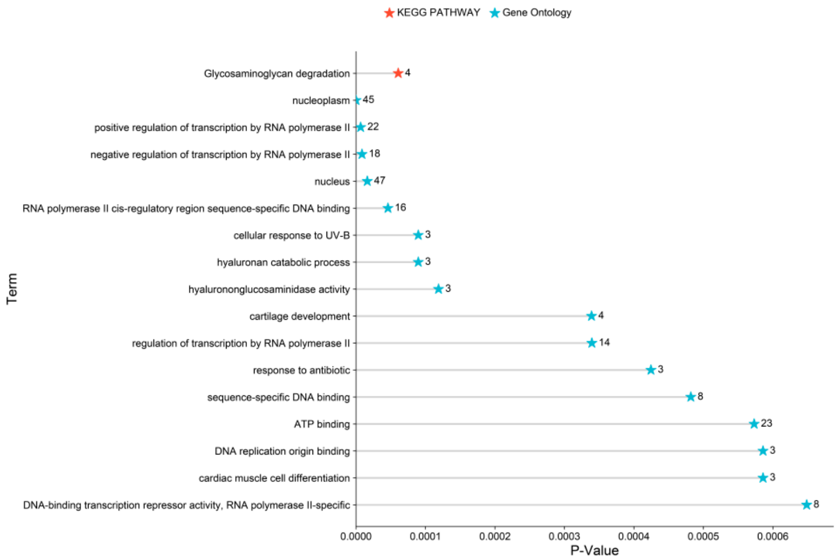

Fig. S4 The KEGG and GO pathways from the genes overlapped in the results iHS and θπ. The The number represents the number of genes.
